# Decellularized matrix grafts and peripheral nerve regeneration

**DOI:** 10.4103/NRR.NRR-D-25-00526

**Published:** 2025-09-19

**Authors:** Qin Zhang, Xingyu Liu, Ye Zhu, Tianmei Qian, Shanshan Wang, Meiyuan Li

**Affiliations:** 1 Key Laboratory of Neuroregeneration of Jiangsu and Ministry of Education, Co-Innovation Center of Neuroregeneration, NMPA Key Laboratory for Research and Evaluation of Tissue Engineering Technology Products, Medical School of Nantong University, Nantong University, Nantong, Jiangsu Province, China; 2 Department of Obstetrics and Gynecology, Affiliated Hospital of Nantong University, Nantong, Jiangsu Province, China; 3 Department of Engineering and Translational Medicine, Tianjin University, Tianjin, China; 4 Engineering Research Center of Integration and Application of Digital Learning Technology, Ministry of Education, Beijing, China

**Keywords:** biological cues, biological scaffold, decellularization, decellularized extracellular matrix graft, decellularized tissue, extracellular matrix, nerve repair and regeneration, peripheral nerve injury, regenerative medicine, tissue engineering

## Abstract

Traditional nerve repair methods, such as autologous nerve grafting and allogeneic nerve grafting, face issues such as donor shortage, functional loss, and immune rejection. Decellularized extracellular matrix-based grafts have emerged as highly promising alternatives, capable of uniquely recreating the natural neural microenvironment, promoting host cell remodeling, and ultimately enhancing functional neural regeneration. This review comprehensively analyzes the key mechanisms of peripheral nerve injury and regeneration, focusing on contemporary therapeutic strategies for key aspects such as axonal apoptosis inhibition, enhanced intrinsic regenerative capacity, construction of regenerative microenvironment, and prevention of target organ atrophy. Findings from this review has shown that decellularized extracellular matrix grafts can promote the migration, proliferation, and differentiation of nerve cells by providing physical support, chemical signals, and mechanical stability. Decellularized extracellular matrix grafts are mainly used as nerve conduits, scaffolds, hydrogels, and 3D printing inks. Decellularized extracellular matrix grafts have demonstrated significant advantages in promoting nerve regeneration by regulating the proliferation and differentiation of Schwann cells, improving the neural microenvironment, reducing inflammatory responses, and promoting angiogenesis. Additionally, decellularized extracellular matrix grafts can serve as drug carriers, enabling the controlled release of growth factors, which further enhances nerve regeneration. However, these grafts also have some limitations, including the presence of immunogenic residues, inadequate mechanical properties, inter-batch variability, and uncontrollable degradation rates. Future research should focus on optimizing the decellularization process, enhancing the mechanical properties of decellularized extracellular matrix grafts, reducing immunogenicity, improving biocompatibility and safety, and developing new composite materials. Furthermore, exploring their application potential in complex nerve injuries, such as diabetic neuropathy, is crucial to meet the needs of peripheral nerve regeneration and repair.

## Introduction

The peripheral nervous system is a fragile yet unprotected tissue, easily affected by various factors such as occupational injuries, vehicular accidents, tumor diseases, and infectious diseases, leading to structural and functional lesions of peripheral sensory nerves, motor nerves, and autonomic nerves. Annually, millions of people worldwide suffer from peripheral nerve diseases (Gu et al., 2011; Yao et al., 2025). Peripheral nerve diseases can be classified in various ways. From the perspective of etiology, they can be categorized into several different types. Nutritional and metabolic diseases, such as those caused by vitamin B deficiency or diabetic peripheral neuropathy, disrupt normal neurological function by disturbing the availability of essential nutrients or through metabolic disorders (Farah and Yammine, 2022; Kramarz et al., 2023). Toxic diseases arise from exposure to harmful substances, including drug poisoning and heavy metal exposure, which can directly damage peripheral nerves (Umapathi and Chaudhry, 2005). Infectious causes, such as bacterial or viral infections, can trigger neuritis and related inflammatory responses within the nervous system. Immune-related diseases, exemplified by Guillain-Barré syndrome, involve an autoimmune attack on peripheral nerve structures. Genetic diseases, such as hereditary motor and sensory neuropathy, result from gene mutations that compromise nerve integrity over time (Grünert et al., 2021; Finsterer, 2022; Latov, 2022). Vasculitis represents another important category, where inflammation of blood vessels affects peripheral nerve function by disrupting blood supply and causing ischemic damage (Kapoor and Reddel, 2024). These diseases can also be classified based on the spatial distribution of nerve damage. Mononeuropathy affects a single nerve, such as in facial nerve palsy, leading to localized functional disorders. Multiple mononeuropathy involves continuous or concurrent damage to multiple individual nerves, resulting in a broader defect. In contrast, polyneuropathy affects most peripheral nerves simultaneously and typically presents as a symmetrical pattern throughout the body. From a pathophysiological perspective, peripheral nerve diseases can be classified according to the primary site of injury. Axonal degeneration is characterized by significant damage to axons, the slender projections of nerve cells responsible for transmitting electrical signals (Stubbs, 2020; Chiang et al., 2022; Kramarz et al., 2023; Schenone et al., 2025). Demyelinating diseases primarily target the myelin sheath of nerves, which insulates axons and promotes effective signal transmission. Mixed diseases combine both axonal and myelin sheath injuries, presenting a more complex pathophysiological picture and often requiring more challenging treatment plans (Motiwala, 2021; Yang et al., 2023c; Tusnim et al., 2024; Wei et al., 2024).

In peripheral nerve disorders, the repair and regeneration of nerves following injury constitute the crux of treatment. Over the past century, numerous studies have documented cases of neural degeneration and attempted regeneration (Fang and Bonini, 2012; Szarek et al., 2013; Stella et al., 2021; Au and Ma, 2022; Dahlin, 2023). However, during the first four decades of investigation, a comprehensive understanding of the regenerative processes eluded researchers. While peripheral nerves possess an intrinsic ability to repair after injury, functional recovery often remains suboptimal. This is especially true in cases involving long-gap nerve defects, where spontaneous regeneration is insufficient and often necessitates surgical intervention (Li et al., 2014). Historically, autologous and allogeneic nerve grafts have been regarded as the gold standards for addressing nerve deficits (Jiang et al., 2010; Yang et al., 2023d). However, autologous nerve grafting involves sacrificing healthy donor nerves and requires a minimum of two surgeries, one at the donor site and another at the recipient site. Its practicality is limited by factors such as the scarcity of available donor nerves, functional loss at the donor site, structural mismatches between donor and recipient sites that may lead to complications, and the risk of nerve tumor formation at the donor site (Gu et al., 2011; Liu et al., 2023b). Allogeneic and xenogeneic nerve transplants, along with other non-neural tissue options such as veins and muscles, frequently encounter challenges related to immunity and antigens (Wong and Griffiths, 2014; Keane and Badylak, 2015; Lans et al., 2023).

Due to the regenerative capacity of the peripheral nervous system, researchers have developed conduits made from natural materials or synthetic polymers. These conduits are sutured or secured to the two ends of the injured nerve to guide axonal migration, maintain nerve growth factors between the distal and proximal stumps, prevent scar tissue invasion, and establish a regenerative pathway for nerve defects (Pinho et al., 2016; Sarker et al., 2018; Lee et al., 2022; Liu et al., 2025a). With the rapid advancement of tissue engineering and regenerative medicine, researchers have begun to combine various types of nerve conduits with scaffolds, cells, and biochemical and physicochemical factors to construct tissue-engineered nerve transplants, guiding regenerating nerve fibers to migrate toward target tissues or organs and achieving functional reinnervation (Klimovich et al., 2021; Escobar et al., 2022; Liu et al., 2022c).

**Figure 1:**
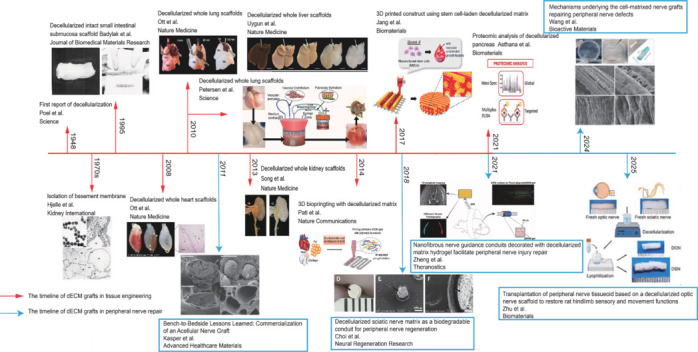
Timeline of dECM grafts. The red arrow indicates the timeline of dECM grafts in tissue engineering (Zhang et al., 2022b). The blue arrow indicates the timeline of dECM grafts in peripheral nerve repair. In 2011, Axogen Corporation designated peripheral nerve allografts as its inaugural product (Kasper et al., 2020). With permission from Advanced Healthcare Materials, copyright 2025, in 2018, a collagen-based nerve conduit containing decellularized sciatic nerve matrix was constructed (Choi et al., 2018). In 2021, a combinatorial neural guidance scaffold consisting of electrospun nanofibers and pDNM gel was fabricated (Zheng et al., 2021). In 2024, a three-dimensional oriented nerve graft based on human bone marrow stem cell-derived dECM was developed (Wang et al., 2024). In 2025, a peripheral nerve tissue composed of a Schwann cell-based NT-3 delivery system and a decellularized optic nerve with natural directional channels was constructed (Zhu et al., 2025). dECM: Decellularized extracellular matrix.

Since the concept of decellularization was introduced, researchers have conducted numerous studies on the application of decellularized extracellular matrix (dECM) grafts in the field of tissue engineering (Zhang et al., 2022c; García-García et al., 2025). In 2011, Axogen Corporation designated peripheral nerve allografts as its inaugural product and acquired relevant technologies, including an optimized decellularization approach obtained from the University of Texas at Austin and a chondroitin sulfate removal technique from the University of Florida (Kasper et al., 2020). In 2018, Choi et al. fabricated a biodegradable, collagen-based nerve conduit containing decellularized sciatic nerve matrix for the repair of sciatic nerve defects in rats. In 2021, Zheng et al. developed a combinatorial neural guidance conduit consisting of longitudinally aligned electrospun nanofibers and porcine decellularized nerve matrix (pDNM) hydrogel, which provided topological and biochemical guidance for directing and promoting axonal extension, myelination of nerve fibers, and functional recovery. Wang et al. (2024b) successfully developed a directional neural graft based on a three-dimensional (3D) matrix by encapsulating dECM derived from human bone marrow mesenchymal stem cells within a fibrous scaffold, achieving multi-layered conformational integrity and biological activity. Recently, Zhu et al. (2025) constructed peripheral nerve tissue bodies composed of Schwann cell-based neurotrophin 3 (NT-3) delivery systems and decellularized optic nerves with natural directional channels. Proteomic analysis and messenger RNA (mRNA) sequencing indicated that peripheral nerve tissue bodies promote nerve regeneration through three mechanisms: chemotaxis, adhesion, and intrinsic mobilization. Ultimately, rapid neural reinnervation and improvement of sensory and motor functions in the hind limbs were achieved (**Figure 1**). The dECM grafts are formed from human or animal organs/tissues through decellularization techniques that eliminate immunogenic cellular components and are primarily composed of the extracellular matrix (ECM), a 3D framework encompassing extracellular macromolecules such as collagen, elastin, fibronectin, laminin, and matrix cell proteins (**Additional Table 1**; Batasheva et al., 2024; Li et al., 2024b; Xu et al., 2024b). The physicochemical signals and biological properties of dECM are preserved, offering a substrate for mechanical support and a biological 3D carrier for subsequent tissue regeneration (Xu et al., 2024a).

**Additional Table 1:** Major protein components of decellularized extracellular matrix

Protein	Distribution	Function	Reference
Collagen	Skin, tendons, ligaments, bones, cartilage, and blood vessels	Provide structural support, maintain the integrity and stability of organ, influence cell behavior and function.	Momot, 2022
Elastin	Aorta, lungs, and some ligaments	Provide flexibility, maintain structure, and regulate mechanical properties.	Vindin et al., 2019; Wen et al., 2020
Fibronectin	Plasma, epithelial tissue, and muscle	Regulate cell behaviors and functions, participate in hemostasis and coagulation, and assist in tissue regeneration and repair.	Dalton et al., 2021; Patten and Wang, 2021
Laminin	Basement membrane	Regulate cell behavior and signal transduction, furnish biologically active sites, and sustain the stability of the extracellular environment.	Kang and Yao, 2022; Mohanty and Roy, 2024
Proteoglycan	Connective tissue, basement membrane, skin	Regulate cell behavior, provide moisture and lubrication, and regulate the activity of growth factors	Valachová et al., 2022; Yuan et al., 2023

The purpose of this review is to summarize the regenerative events that occur after nerve injury and to discuss strategies for promoting the recovery of damaged nerve function. It focuses on the current research status of cell-matrix tissue-engineered nerve transplantation. Specifically, this review examines the regenerative processes following nerve injury and highlights strategies that can enhance recovery, with a particular emphasis on the application prospects of cell-matrix nerve grafts in peripheral nerve regeneration. This paper provides an in-depth clarification regarding the mechanisms underlying nerve regeneration, thereby laying a foundation for a better understanding of the pathophysiological processes associated with peripheral nerve diseases. Additionally, this review summarizes new treatment strategies, opens new avenues for addressing peripheral nerve disorders, with the purpose of enhancing therapeutic outcomes. Findings from this review will further promote the research and development of biomaterials, optimizing and innovating cell-free matrix grafts to meet clinical needs. This review emphasizes the importance of multidisciplinary collaboration, advocating for the integration of knowledge and technologies from materials science, neurobiology, and clinical medicine to effectively address the challenges associated with treating peripheral nerve diseases.

## Search Strategy

Studies cited in this review were published from 1948 to 2025, with a predominant citation from 2022 to 2024. All studies cited here were searched on PubMed database using search terms: “peripheral nerve injury” OR “nerve repair and regeneration,” “biological cues,” “biological scaffold” OR “tissue engineering,” “regenerative medicine” OR “regenerative microenvironment,” “tissue-engineered nerve grafts”, “decellularization techniques” OR “decellularization methods,” “extracellular matrix components” OR “extracellular matrix functions,” “decellularized matrix nerve grafts.” The results were further filtered by title/abstract; non-SCI experimental and review articles were excluded. After removing duplicates from the retrieved studies, we first examined the titles and abstracts of each article as a preliminary screening measure. We then conducted a thorough review of the full texts to exclude studies that did not address peripheral nerve regeneration and dECM grafts. Ultimately, 393 references were included in this review.

## Mechanisms of Peripheral Nerve Regeneration and Therapeutic Strategies

**Figure 2:**
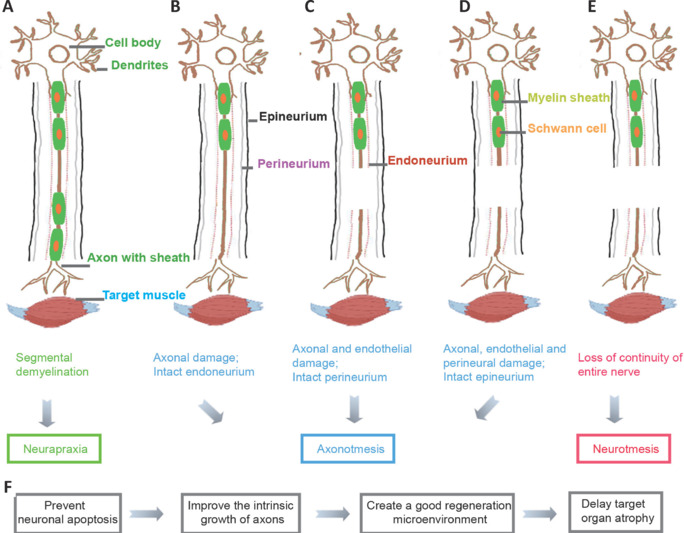
Classification of peripheral nerve injury (PNI) and regeneration strategies. (A) First-degree injury (neurapraxia), (B) second-degree injury (axonotmesis), (C) third-degree injury (axonotmesis), (D) fourth-degree injury (axonotmesis), and (E) fifth-degree injury (neurotmesis). (F) The main strategies for regenerating peripheral nerves.

After peripheral nerve injury (PNI), the nerve and surrounding connective tissues respond as part of a complex network. This injury can be broadly categorized into two types: non-degenerative and degenerative. Non-degenerative PNI refers to an injury without axonal loss, whereas degenerative PNI involves axonal damage. PNI can be further classified into three major categories: neurapraxia, axonotmesis, and neurotmesis (Lopes et al., 2022). In neurapraxia, the neural structure remains intact, but its ability to transmit impulses is lost. In axonotmesis, the axon is damaged or destroyed while the surrounding connective tissue remains intact. Neurotmesis involves damage to both the nerve trunk and the surrounding connective tissue (Campbell, 2008; Barnes et al., 2022). Sunderland (1990) further classified axonotmesis into three categories based on varying levels of nerve damage and potential for spontaneous nerve regeneration. According to Sunderland, neurapraxia is classified as first-degree PNI, characterized by segmental demyelination where the axons remain structurally unharmed. However, destruction of the myelin sheath renders the axons incapable of transmitting impulses, resulting in paralysis of the affected area. Axonotmesis can be categorized into second-, third-, and fourth-degree PNI based on the extent of damage to other structures within the peripheral nerve. Second-degree PNI involves axonal damage while retaining the endoneurium, allowing for optimal regeneration. Third-degree PNI presents with damage to the axon and endoneurium, while the perineurium and fascicular plexus remain intact. Fourth-degree PNI includes injury to the axon, endoneurium, perineurium, and fascicular pattern, but the epineurium is spared. Neurotmesis, classified as fifth-degree PNI, involves injury to the entire nerve trunk with a loss of continuity (Jiang et al., 2020; Vijayavenkataraman, 2020; **Figure 2**). A significant event following PNI is Wallerian degeneration, which is characterized by swelling of the distal neuron cell body, retrograde changes in axons, destruction of the myelin sheath, and activation of Schwann cells (Nocera and Jacob, 2020; Slavin et al., 2021; Gu et al., 2024). The advancement of the growth cone from the proximal nerve axon marks the beginning of nerve regeneration, a complex process regulated by multiple factors (Chen et al., 2015; Bosch-Queralt et al., 2023). Understanding the cellular and molecular mechanisms involved in peripheral nerve regeneration has become a central focus of contemporary neural regeneration research. Current strategies for promoting peripheral nerve regeneration include preventing neuronal apoptosis, enhancing the intrinsic growth capacity of axons, establishing a favorable regenerative microenvironment, and delaying the atrophy of target organs (O’Brien et al., 2022; Bosch-Queralt et al., 2023; Mankavi et al., 2023; Sarhane et al., 2023; **Figure 2**).

### Inhibiting neuronal apoptosis and furnishing a driving force for regeneration

After peripheral nerve damage, neurons are susceptible to several detrimental factors, including retrograde injury, neurotrophic factor deficiency, excitotoxicity, and oxidative stress-mediated cell death. The death of neuronal cells releases harmful substances, which intensify local inflammation and promote the proliferation of glial cells, thereby creating a toxic environment for regeneration. Therefore, controlling neuronal cell death is vital (Zhang et al., 2022a).

Apoptosis can be detected in neurons within the first week after injury, with the rate of apoptosis in dorsal root ganglion (DRG) neurons reaching 35%–40% by the second month post-injury. The regulation of neuronal apoptosis depends on a delicate balance between pro-survival proteins and pro-apoptotic proteins (Terenghi et al., 2011). One important molecule involved in neuronal survival is growth arrest and DNA damage-inducible 45 alpha (GADD45α). Upregulation of GADD45α expression in DRG neurons after injury can protect against apoptosis, while downregulation of this gene increases neuronal cell death (Lin et al., 2011). PNI induces a series of morphological, molecular, and gene expression changes in spinal cord and DRG neurons, some of which can enhance neuronal survival and regenerative capacity (Lyu et al., 2020; Gu et al., 2022; Feng et al., 2023). To leverage these beneficial changes, researchers have implemented proactive strategies to counteract the negative effects of peripheral nerve damage. The administration of neurotrophic factors, acetyl-L-carnitine, N-acetylcysteine, and the NMDA receptor antagonist MK-801 has been shown to alleviate axonal severation-induced neuronal apoptosis in a time-dependent manner and promote neural regeneration. Additionally, Scholz et al. (2005) focused on suppressing the activation of cysteine-aspartic acid proteases (caspases) to inhibit apoptosis in dorsal horn neurons induced by nerve injury. This approach can sustain inhibitory transmission in lamina II and alleviate pain hypersensitivity.

### Enhancing the intrinsic axonal growth ability by modulating transcription factors, growth cone protein translation, and mitochondrial metabolism

Before transcriptional changes occur in injured neurons, peripheral nerves relay injury signals retrogradely to the neuronal cell body (Lu et al., 2012; Villard, 2023). Studies using a rat sciatic nerve transection model have revealed that retrograde signaling is mediated by input proteins that form complexes with cytoskeletal proteins (Rishal and Fainzilber, 2014; Yamashita, 2019; Tomé and Almeida, 2024). These complexes subsequently bind to transcription factors and are transported to the cell nucleus, establishing a signaling pathway from the injury site to the neuronal soma (Hanz et al., 2003; Wu et al., 2024). To promote a rapid response to injury, these input proteins and transcription factors, including activating transcription factor 3 (ATF3), signal transducer and activator of transcription, and nuclear factor of activated T cells, are locally translated in damaged axons following injury (Patodia and Raivich, 2012). In healthy DRG neurons, the expression level of ATF-3 is low, but it is rapidly upregulated after injury. Overexpression of ATF-3 in cultured DRG neurons has been shown to enhance axonal growth, suggesting the crucial role of this transcription factor in nerve regeneration (Cheng et al., 2021). Heat shock protein 27 (Hsp27) is one of the proteins regulated by ATF-3. Transgenic mice that overexpress Hsp27 exhibit a higher proportion of axonal regeneration following sciatic nerve crush injuries and demonstrate a reduced incidence of neuronal apoptosis, leading to improved functional recovery during regeneration (Zhou et al., 2019b; McCormick and Gupton, 2020).

The translation of proteins on the growth cone of neurons is crucial for promoting extensive axonal growth. Z-DNA-binding protein 1 binds to β-actin mRNA and plays a critical role in local β-actin translation and growth cone guidance (Lepelletier et al., 2017). In addition, inhibiting the expression of Z-DNA-binding protein 1 on the growth cone limits mRNA localization and neural regeneration, highlighting the important role of local protein translation in the intrinsic regeneration process of neurons. The speed of axonal regeneration can be improved through translation localization on the growth cone (Jones et al., 2021; Kong et al., 2022).

Mitochondria, as the main energy source for cellular metabolism, are crucial for neuronal development and synaptic transmission (Persson et al., 2016; Yang et al., 2021a). Mitochondria provide necessary protection for damaged peripheral nerve cells by accelerating mitochondrial transport, reducing energy deficiency, minimizing cytoskeletal damage, and eliminating harmful reactive oxygen species (Nicholls and Budd, 2000; Fairley et al., 2022; Iwata et al., 2023). Therefore, strategies targeting axons to enhance mitochondrial transport capacity, such as overexpression of proteins including Armcxl and Miro, or reducing mitochondrial transport resistance by knocking out factors, such as SNPH, can significantly enhance the regenerative ability of nerve cells and provide a more solid foundation for the recovery of neural function (Adebayo et al., 2021). Meanwhile, the transportation and translation of mRNA in axons are of paramount significance for the viability of neurons. The local synthesis of nuclear-encoded mitochondrial proteins can protect long-lived axonal mitochondria from damage (Zaninello et al., 2024).

### Maintenance functions of Schwann cells, immune cells, extracellular matrix, cell adhesion molecules, neurotrophic factors, and exosomes in the regenerative microenvironment

After PNI, a specialized cellular and molecular microenvironment rapidly evolves, encompassing elements such as inflammatory responses, ECM components, neurotrophic factors, and blood supply. These factors work synergistically to provide essential support and stimulation for the regeneration and recovery of neurons (Marquardt and Sakiyama-Elbert, 2013; Chen et al., 2015; Ren et al., 2024; Wei et al., 2024).

#### Schwann cells in Wallerian degeneration

Following PNI, the disruption of axonal interactions prompts both myelinating and non-myelinating Schwann cells to transition from a mature state to a proliferative, precursor-like “repair” state, a process termed “dedifferentiation.” This transformation reactivates the expression of developmental genes that are essential for nerve repair (Stratton et al., 2018; Li et al., 2020c; Bosch-Queralt et al., 2023; Xu and Fan, 2025). Nerve growth factor (NGF) activates the autophagic signaling pathway in Schwann cells to enhance the clearance of myelin fragments and accelerate nerve regeneration. Meanwhile, the downregulation of myelin protein expression promotes the upregulation of growth-related genes, including those encoding cell adhesion molecules, growth factors, and their receptors (Kister and Kister, 2022; Martinsen and Kursula, 2022). The changes in Schwann cells following injury may be actively triggered by injury signals or may result from the loss of axonal signals (Willem et al., 2006; Ling et al., 2023). Neuroregulin 1 is one of the most typical axonal signals. When the type III isoform of neuroregulin 1 is cleaved by β-secretase 1 and interacts with the ErbB receptor family on Schwann cell membranes, it induces their proliferation and myelin production. Silent information regulator 6 functions as a negative regulator of Schwann cell dedifferentiation during Wallerian degeneration, whereas c-Jun acts as a direct downstream effector of silent information regulator 6 in injured peripheral nerves (Zou et al., 2022). Another factor that may influence Schwann cell function is the Notch transmembrane receptor, where the cleavage and nuclear translocation of its intracellular domain increase Schwann cell proliferation and negatively regulate myelin production. In the distal nerves after injury, the increased expression of nuclear translocation of its intracellular domain in Schwann cells leads to faster dedifferentiation (Woodhoo et al., 2009; Liu et al., 2025b).

The transcription factor c-Jun, a component of the activator protein-1 complex, plays a crucial role in regulating the phenotype of Schwann cells. Following injury, c-Jun is upregulated in Schwann cells involved in the repair process. When c-Jun is inactivated in these cells, the number of Schwann cells involved in the repair process decreases, resulting in the persistence of myelin fragments, disruption of bands of Büngner, impaired recovery of neural function, and inhibition of neural regeneration (Jessen and Mirsky, 2021; Gao et al., 2022b; Xu et al., 2023). Culturing Schwann cells in dishes containing ECM components has been found to lead to high expression of c-Jun, indicating that ECM components mediate the regulation of Schwann cell morphology, proliferative capacity, and protein expression (Xu et al., 2020). Additionally, certain microRNAs (miRNAs) are associated with the phenotype, proliferation, and migration of Schwann cells. For instance, the double-negative feedback loop between miRNA-363-5p and the P2X receptor subunit 4 facilitates the dedifferentiation and migration of Schwann cells after nerve injury (Sohn et al., 2021). Conversely, miR-148b inhibits the proliferation and migration of Schwann cells through calreticulin (Zhou et al., 2019a). Furthermore, the internalization of endothelial exosomes in Schwann cells can enhance neural regeneration by promoting and maintaining repair-related phenotypes in these cells, which may be linked to the upregulated expression of miR-199-5p and the activation of the PI3K/AKT/PTEN signaling pathway (Huang et al., 2023).

#### Immune regulatory response

In the distal neural environment, in addition to the involvement of Schwann cells, axonal debris and myelin fragments are phagocytosed by resident macrophages or macrophages recruited through inhibitory factors secreted by Schwann cells, thereby laying the foundation for distal regeneration (Zigmond and Echevarria, 2019; Wu et al., 2023a). The role of macrophages is pivotal in peripheral nerve regeneration, with their phenotype significantly impacting regenerative outcomes: classically activated (M1) macrophages promote inflammation, whereas alternatively activated (M2) macrophages exert anti-inflammatory effects (Mueller et al., 2003; Huang et al., 2020; Jha et al., 2021). Most microglia and recruited macrophages in the central nervous system exhibit an M1 phenotype and continuously provide pro-inflammatory signals at the injury site. In contrast, the recruited macrophages at PNI sites tend to have a relatively higher proportion of the M2 phenotype, which helps restrain inflammation while promoting repair. Inducing a shift in phenotype from destructive M1 macrophages to protective M2 macrophages using anti-inflammatory cytokines may be an effective approach to promote peripheral nerve regeneration. In addition to clearing inhibitory debris, oriented microfibers can induce macrophage polarization to guide Schwann cell activation (Dong et al., 2021; Li et al., 2025a). Furthermore, macrophages produce growth factors and regulate ECM components, coordinating the transmission of regeneration signals (Saio et al., 2021; Le et al., 2024). Macrophages sensing hypoxic conditions can also produce and release vascular endothelial growth factor (VEGF)-A, guiding endothelial cell proliferation and vascular formation through chemotactic signals, thereby providing a pathway for Schwann cell migration (Cattin et al., 2015; Fan et al., 2024). After sciatic nerve injury, cathepsin S derived from M2 macrophages cleaves Ephrin-B2 in fibroblasts, which then binds to EphB2 in Schwann cells, activating Schwann cells and promoting axonal regeneration (Oshima et al., 2023).

#### Extracellular matrix and cell adhesion molecules

After PNI, Schwann cells rapidly proliferate and synthesize and secrete various neurotrophic factors, ECM components, inflammation-regulating factors, and adhesion molecules (Bosch-Queralt et al., 2023). The ECM typically consists of five types of substances: collagen, non-collagen proteins, elastin, proteoglycans, and glycosaminoglycans (GAGs). These matrix components interact with each other, bind to cell adhesion receptors, and collectively form a complex network (Bolívar et al., 2020; Sutherland et al., 2023). Laminin and fibronectin play crucial roles in Schwann cell adhesion, migration, axon guidance, growth factor signaling, and the regulation of cell function. The ECM has a dual regulatory effect on axons. In undamaged nerves, chondroitin sulfate inhibits neuronal cell growth and axonal extension to prevent axonal branching at the node of Ranvier, thereby promoting precise nerve innervation and maintaining nerve function. However, after nerve injury, laminin plays an important role in promoting axon growth and chemotactic regeneration (Liu et al., 2010; Vahidi et al., 2024). Additionally, the ECM forms an ordered and dense reticular basement membrane near the cell membrane, providing a scaffold for axonal extension. Matrix metalloproteinases (MMPs) exert hydrolytic actions to dynamically remodel the ECM, diversifying its functions. Membrane type 1-MMP (MT1-MMP), MMP-2, and MMP-9 promote axon growth and repair by removing glial scars from injured areas (Remacle et al., 2018). Furthermore, these MMPs also play a significant role in the formation and functional regulation of neuronal synapses. For instance, synaptic elimination can be achieved by hydrolyzing or degrading growth factors, neurotrophic factors, and ECM molecules, thereby optimizing the formation and function of neural circuits (Chan et al., 2020; He et al., 2022; Asgari et al., 2023).

Cell adhesion molecules, including the immunoglobulin superfamily, integrins, and selectins, are a class of proteins present on the cell surface. They play a crucial role in the mutual recognition, adhesion, and signal transduction between cells, as well as between cells and the ECM, influencing the process of nerve cell regeneration and reconstruction (Zhang et al., 2008; Lavdas et al., 2011; Kong et al., 2022). Studies have shown that Schwann cells express different subtypes of integrin subunits on their cell surface (Previtali et al., 2001; Su et al., 2008). After nerve injury, Schwann cells induce the expression of α6 and β1 integrins, which may play a role in mediating the interaction between Schwann cells and axons and promoting axon regeneration (Chang et al., 2018).

#### Neurotrophic factors

Neurotrophic factors (NTFs) are typically produced by neurons and other cells, regulated by factors such as neuronal activity, synaptic transmission, and neuronal interactions. Key examples of NTFs include NGF, brain-derived neurotrophic factor, NT-3, and neurotrophin-4/5. These polypeptide growth factors primarily act on the nervous system, influencing the growth, differentiation, survival, synaptic formation, and functional maintenance of neurons and glial cells. They are essential for maintaining a regenerative microenvironment (Boyd and Gordon, 2003; Lien et al., 2020; Zhang et al., 2022b). NTFs activate intracellular signaling pathways by binding to specific receptors on the surface of neurons, leading to a series of biological effects. Currently, three types of receptors associated with NTFs have been identified: TrkA (which binds to NGF), TrkB (which binds to brain-derived neurotrophic factor), and TrkC (which binds to NT-3) (Becker et al., 2018; Gonçalves et al., 2020). In response to nerve damage, these NTFs can be rapidly released to regulate the inflammatory response, promote the formation of new blood vessels, supply nutrients and oxygen, and create a microenvironment conducive to nerve regeneration and functional recovery. Researchers are increasingly exploring the therapeutic potential of NTFs and their associated receptors (Huang et al., 2019; Baltrusch, 2021).

#### Exosome

Exosomes originate from the endoplasmic reticulum and the multivesicular body system, emerging through the fusion of the endoplasmic reticulum and Golgi apparatus to form small extracellular vesicles with diameters ranging from approximately 30 to 150 nm. Rich in biomolecules, exosomes are key tools for intercellular communication, regulating the growth, survival, and function of neurons through the transfer of bioinformatic molecules (Wong et al., 2022; Supra and Agrawal, 2023). The miRNAs and protein-active factors they convey play crucial roles in maintaining cellular homeostasis (Qing et al., 2018). Research indicates that neurons, glial cells, and neural stem cells are capable of secreting exosomes, which play a critical role in synaptic growth and the repair of damaged nerves (Li et al., 2023a; Dogny et al., 2024). Exosomes derived from Schwann cells are enriched with miRNAs that regulate neuronal growth and can inhibit the activity of the GTPase Rho, which, when activated, leads to growth cone collapse. This inhibition facilitates the recovery of peripheral nerves (Li et al., 2017). Exosomes derived from bone marrow stem cells (BMSCs) possess characteristics of analgesia, anti-inflammation, and tissue regeneration. Researchers have designed a conductive nerve dressing containing BMSCs-derived exosomes (BMSCs-Exos) and found that it has significant potential for nerve regeneration, functional restoration, and pain relief in patients with diabetic PNI (Yang et al., 2023b). NTN1^+^ endothelial cells (NTN1 + ECs) constitute a crucial component of the vascular microenvironment. The NTN1 + ECs-derived exosomes (NTN1 EC-EXO) carry a low expression level of let7a-5p and activate key pathways associated with microenvironment formation, such as focal adhesion, axon guidance, phosphatidylinositol 3-kinase-AKT, and mammalian target of rapamycin signaling pathways, thereby establishing a beneficial microenvironment for nerve repair (Huang et al., 2024). Platelet-rich plasma-derived exosomes contain abundant bioactive molecules that can significantly enhance the proliferation, survival, and migration ability of mesenchymal stem cells (MSCs), reduce MSC apoptosis under stress, maintain the stem cell characteristics of MSCs, and alleviate MSC aging. They may represent a novel type of treatment that increases MSCs’ ability to promote nerve repair and regeneration (Zhang et al., 2024b).

### Slowing the progression of denervated atrophy in target muscle

Over time, chronic denervation of muscles leads to noticeable changes, including a decrease in the number of muscle fibers, which negatively affects the proliferative capacity of satellite cells. When the remaining muscles are reinnervated, although synaptic structures of neuromuscular junctions can form, muscle function does not fully recover (Kostrominova, 2022). In chronic denervation animal models associated with poor functional recovery, it is observed that regenerating nerve axons near the target muscle’s motor endplates exhibit small branches, thereby failing to form complete synapses (Ma et al., 2011). To promote nerve regeneration, two important aspects need to be considered. First, accelerating axonal extension speed is crucial to ensure that axons reach the target location as quickly as possible. Transgenic mice with Hsp27 exhibit rapidly growing axons that can reach their target organs during critical periods, forming functional synapses with chronically denervated muscles (Höke, 2011). Second, implementing appropriate measures to slow down the progression of target muscle denervation can provide valuable time for nerve regeneration and increase the chances of target muscles being reinnervated. Xu et al. (2021) found that the expression of muscle mitochondrial peroxiredoxin prevents nerve-muscle junction disruption and atrophy in a mouse model of muscle cell loss. Additionally, anti-oxidative stress protein 2 protects denervated muscles from atrophy through the unfolded protein response and mitochondrial engulfment (Yang et al., 2021b), while GADD45α acts as a protective regulator against neurogenic muscle atrophy, demonstrating good protective effects against denervation atrophy (Ehmsen et al., 2021). It is assumed that inhibition of histone deacetylase 4 will mitigate muscle atrophy induced by denervation (Ma et al., 2021). Recently, a new long non-coding RNA Mir22hg has been discovered, which inhibits histone deacetylase 4 by generating miR-22-3p. In muscle, down-regulation of Mir22hg significantly reduces muscle mass and delays the muscle regeneration and repair processes after injury (Li et al., 2021a).

## Novel Tissue Engineering Concepts for Promoting Neural Function Recovery via the Decellularized Extracellular Matrix Graft Strategy

Tissue engineering and regenerative medicine constitute a rapidly evolving interdisciplinary field that synthesizes principles from life sciences, materials science, and bioengineering. This field is dedicated to the development and fabrication of tissue-engineered grafts designed for transplantation into the human body, with the aim of replacing severely damaged or absent tissues and organs (Jahromi et al., 2019; Cosgriff-Hernandez and Timmins, 2022; Gao et al., 2022a). A prototypical tissue-engineered nerve graft is composed of scaffold materials that deliver biochemical signals through supporting cells and growth-inducing factors, including growth factors, cytokines, and chemotactic factors, thereby promoting nerve repair and regeneration (Phamornnak et al., 2022).

Although tissue engineering and regenerative medicine have demonstrated the potential of using engineered biomaterials and grafts to repair and regenerate tissues and organs, current structures face limitations in replicating complex natural microenvironments and achieving optimal regeneration and functional recovery (Elkhenany et al., 2022; Yadav et al., 2024). To address these issues, the utilization of decellularized tissues and cell-derived ECMs has emerged as a promising solution. Decellularized, biocompatible, and bioactive materials can be prepared into porous scaffolds and grafts that simulate the structure, composition, and properties of the natural tissue or organ microenvironment both *in vitro* and *in vivo* (Snyder and Jana, 2022; Zhang et al., 2023a). The primary components of decellularized ECM (dECM) grafts include ECM molecules such as collagen, elastin, fibronectin, and various growth factors. The analysis of dECM grafts primarily focuses on these key ECM components, which can be identified through quantitative and qualitative biochemical and molecular biological methods, including immunohistochemistry, mass spectrometry, polymerase chain reaction, and western blotting. dECM grafts with biological activity can create individualized tissue-specific microenvironments that tightly control and direct cell behaviors, substantially enhancing therapeutic efficiency for regenerative and repair processes (Brown et al., 2022; Liu et al., 2023a; Long et al., 2023; Batasheva et al., 2024).

### Components of the decellularized extracellular matrix grafts and their individual applications in neural regeneration

**Figure 3:**
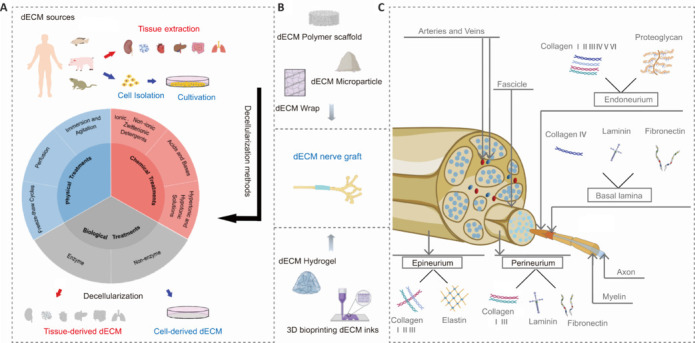
Advanced tissue engineering approaches for enhancing neural function recovery through the dECM graft strategy. (A) The origin and production method of dECM grafts; (B) various applications of dECM grafts in the repair of peripheral nerve defects; (C) the functions of various components in dECM grafts for peripheral nerve regeneration. The epineurium of peripheral nerves is predominantly constituted by collagen (such as type I and type III collagen), proteoglycans, and elastin. The perineurium is abundantly endowed with collagen (such as type I and type III collagen), fibronectin, and proteoglycans. The endoneurium encompasses collagen (such as type III collagen), proteoglycans, and a minute quantity of fibronectin. The basement membrane is primarily composed of type IV collagen, laminin, and heparan sulfate proteoglycans. dECM: Decellularized extracellular matrix.

In the construction of nerve grafts, the commonly used natural materials include ECM components such as collagen, laminin, fibronectin, GAGs, and elastin (**Additional Table 1**). The epineurium of peripheral nerves primarily consists of collagen (including types I and III), proteoglycans, and elastin. The perineurium is rich in collagen (including type I and type III), fibronectin, and proteoglycans. The endoneurium contains collagen (specifically type III), proteoglycans, and a small quantity of fibronectin. The basement membrane is mainly composed of type IV collagen, laminin, heparan sulfate proteoglycan, and other components (**Figure 3C**). These ECM components collectively create a microenvironment conducive to the survival and function of nerve cells, playing an indispensable role in maintaining the normal physiological functions of nerves and promoting nerve regeneration and repair after injury (Li et al., 2022b; Yu et al., 2023).

#### Collagen

Collagen, the most abundant protein in the body, exhibits significant diversity at both the molecular and supramolecular levels. Each collagen subtype can assemble and integrate with various non-collagenous molecules to form complex functional structures (Momot, 2022). Collagen is widely used to prepare different forms of nerve conduits for repairing peripheral nerve defects. Compared with rats repaired with polyglycolic acid nerve conduits, those treated with collagen nerve conduits demonstrated improved isometric muscle contractility, axon count, wet-to-weight ratio of target muscle, and axonal spastic tissue (Waitayawinyu et al., 2007; Liu et al., 2022d). Ordered collagen fibers can guide axonal growth and regulate Schwann cell phenotypes, making them effective internal fillers in the construction of tissue-engineered nerve grafts (Gnavi et al., 2015; Trueman et al., 2024). Chitosan/collagen composite scaffolds, through dual biofunctionalization, promote both endothelial cell proliferation and vascularization, as well as Schwann cell proliferation and nerve regeneration (Li et al., 2020a; Yun et al., 2024). Poly(lactic-co-glycolic acid (PLGA) sheaths and collagen membranes have been shown to exhibit stage-wise degradability and excellent biocompatibility. These conduits are fabricated by electrospinning PLGA membranes, while the intraluminal filler is obtained by freeze-drying a collagen membrane that resembles the ECM (Hou et al., 2023).

#### Elastin

Elastin is a precursor protein monomer with a molecular weight of 60–70 kDa, synthesized and secreted by various cells. These protein monomers are randomly organized and interconnected to form a net-like structure, which gives tissues a certain level of stretchability and elasticity to accommodate different physiological activities and tissue deformations (Vindin et al., 2019; Trębacz and Barzycka, 2023; Gardeazabal and Izeta, 2024). In its natural state, elastin possesses mechanical stability, elasticity, and biological activity, and it is capable of self-assembly, making it a promising material for the development of multifunctional biomaterials (Wen et al., 2020; Szychowski et al., 2022). By utilizing customized elastin-derived peptide sequences, the structure, physical, and chemical properties of elastin-based materials, as well as the desired tissue characteristics, can be tailored, providing a wide range of applications. Additionally, elastin and elastin-like peptides can be modified or combined with other natural or synthetic materials to enhance their functions or impart new structural and biological properties (Almine et al., 2010). Elastin-derived constructs exhibit excellent tunability and may find applications in biomedical and tissue engineering fields, including drug targeting, cell encapsulation, vascularization, nerve regeneration, wound healing, and the replacement of skin, cartilage, bone, and teeth (Wen et al., 2020; Milligan et al., 2022; Vindin et al., 2022). In rat sciatic nerve injury models, recombinant elastin-like peptide (ELP) hydrogels were evaluated as a bridging interstem cellular population. Compared with bridging a 10 mm nerve gap with a hollow silicone conduit, ELPs significantly increased the likelihood of filling the two distal nerve stubs with bridging tissue (Suhar et al., 2021).

#### Fibronectin

Fibronectin is assembled by cells into viscoelastic fibers that can bind up to 40 distinct growth factors and cytokines. These fibers play a crucial role in the provisional ECM assembly during embryonic development and wound healing. The assembly of fibronectin fibers is often upregulated in disease conditions, including cancer and fibrotic disorders. The unique mechanical properties of fibronectin fibers enable them to modify the mechanotransduction signals perceived and relayed by cells. When soluble growth factors bind to fibronectin fibers, they alter the signal transduction of these proteins. Additionally, the binding of other ECM proteins, such as collagen, elastin, and proteoglycans, to fibronectin fibers facilitates ECM maturation and tissue specificity (Dalton and Lemmon, 2021; Patten and Wang, 2021; Gimeno et al., 2022; Ahn et al., 2024; Longstreth and Wang, 2024). Fibronectin can regulate Schwann cell migration, adhesion, and neuronal growth (Mukhatyar et al., 2011; Torres-Mejía et al., 2020). By coupling recombinant fibronectin fragments (FNIII9*-10/12-14) to a polyethylene glycol scaffold, researchers can provide cell adhesion and growth factor binding domains in a 3D environment, thereby promoting neuronal growth (Licht et al., 2019).

#### Laminin

Laminin, through its binding to molecules such as type IV collagen and heparan sulfate, provides attachment sites essential for tissue homeostasis and plays a significant role in basement membrane assembly (Kang and Yao, 2022; Mohanty and Roy, 2024). It also regulates Schwann cell proliferation, differentiation, myelination, and axon regeneration (Yu et al., 2007). A helical nerve graft made from polycaprolactone incorporates laminin onto aligned nanofibers. PC-12 cell assays have confirmed that laminin-functionalized nanofibers can effectively guide and enhance axonal growth (Chang et al., 2020). Additionally, laminin-modified poly(3-hydroxybutyrate-co-3-hydroxyvalerate)/polyethylene oxide nanofiber nerve conduits maintain the beneficial properties of laminin and can be used to repair 12 mm sciatic nerve defects in rats, promoting directed nerve growth (Zhang et al., 2018). In another study, when Schwann cells and neural stem cells were co-transplanted into laminin-chitosan-PLGA nerve conduits for repairing 5 mm recurrent laryngeal nerve injuries in SD rats, optimal regeneration outcomes were achieved (Li et al., 2020). A series of multi-channel nerve conduits were fabricated using longitudinally aligned laminin-coated PLGA/carbon nanotube nanofibers as the inner lumen, while the outer surface was made of randomly oriented polycaprolactone. This design facilitated cell adhesion and proliferation on both the nanofibers and fibrin gel (Nazeri et al., 2022). Furthermore, scaffolds were created by electrospinning two natural polymers, polyhydroxybutyrate and gelatin, with laminin coated on the surface to promote the growth of nerve cells (Zamanifard et al., 2023).

#### Proteoglycans

Proteoglycans are distributed within the collagen fibers of various ECMs, imparting properties that extend beyond mere structural strength. These glycoproteins carry GAGs, which, due to their high negative charge, adopt an extended conformation. This conformation allows them to attract divalent cations, such as water and calcium ions, filling space and providing lubrication (Hao et al., 2022; Nguyen and Panitch, 2022). Hyaluronic acid is a non-sulfated glycosaminoglycan that promotes cell proliferation and migration while facilitating wound healing (Tang et al., 2024; Li et al., 2025b; Zheng et al., 2025). Its viscoelasticity, physiological activity, and biocompatibility make it an ideal material widely used in tissue engineering (Valachová et al., 2022; Iaconisi et al., 2023; Yuan et al., 2023). Tissue-engineered nerve conduits containing hyaluronic acid hydrogel particles effectively promote both morphological and functional recovery of damaged sciatic nerves (Yang et al., 2023a). Hyaluronic acid is often used in conjunction with other materials. For instance, the hyaluronic acid-doped poly(3,4-ethylenedioxythiophene)/chitosan/gelatin (PEDOT-HA/Cs/Gel) porous conductive scaffold demonstrates higher cell adhesion efficiency and viability compared with other conductive scaffolds, as well as elevated expression levels of synaptic growth genes such as GAP43 and SYP (Wang et al., 2017). Additionally, it can be prepared as an injectable biodegradable hydrogel in combination with chitosan, or as a degradable synthetic polymer alongside PLGA and poly-L-lysine, for the repair of peripheral nerve injuries (Wang et al., 2011b). A photothermal material, polydopamine nanoparticles@hyaluronic acid methacryloyl hydrogel (PDA NPs@HAMA), has also been synthesized. In a rat sciatic nerve adhesion model, the photothermal effect of PDA NPs@HAMA protected the nerve from adhesion, thereby preserving nerve function and effectively preventing adhesion-related impairments (Zhan et al., 2023).

#### Extracellular matrix–bound growth and secreted factors

Growth factors that bind to ECM proteins are considered major constituents of the ECM itself (Roytman et al., 2025). Research has shown that growth factors and secreted factors often interact with GAGs, particularly heparan sulfate (Bishop et al., 2007; Hayashida et al., 2022). However, under certain conditions, these factors can also interact directly with specific domains of ECM proteins. For instance, fibronectin has the ability to bind various growth factors, including VEGF, hepatocyte growth factor, and platelet-derived growth factor (Kim et al., 2025). It has also been extensively studied for its binding to bone morphogenetic proteins through its von Willebrand factor type C and chondroitin sulfate domains, which are commonly found in numerous ECM proteins (Badylak, 2004; Ferguson et al., 2020). Transforming growth factor selectively binds to the latent transforming growth factor beta binding protein via its TB domain and is further associated with the fibrin/fibronectin-rich ECM (Peng et al., 2022). The ECM serves not only as a storage reservoir and regulatory platform for these factors, but it also plays a crucial role in development. For example, chemokines and signaling molecules such as VEGF, Wnt proteins, hedgehog proteins, bone morphogenetic proteins, and fibroblast growth factors are essential for embryonic development. These molecules can be organized into concentration gradients within tissues, regulating developmental patterns. The formation of these gradients is closely linked to their attachment to the ECM (Wu et al., 2016; Guo et al., 2021; Liu et al., 2022b). Additionally, the ECM regulates the retention and presentation of growth factors through electrostatic interactions and other mechanisms, such as integrin aggregation, which influences growth factor signaling. Therefore, synthetic materials or ECM-derived proteins and tissues, when used in conjunction with other biomaterials, can mimic the properties of the ECM and provide growth factors for tissue regeneration (Gresham et al., 2021).

### Decellularization methods for the fabrication of decellularized extracellular matrix grafts

Qualified dECM scaffolds are characterized by residual cellular content that adheres to specific quantitative criteria (Crapo et al., 2011; Neishabouri et al., 2022; Salti et al., 2023). Key benchmarks include a DNA content of less than 50 ng per mg of dry weight, DNA fragment lengths under 200 base pairs, and an absence of visible nuclear material when assessed using tissue staining techniques, such as hematoxylin and eosin staining. Currently, there is no universally accepted methodological gold standard for decellularization; the process is highly dependent on various attributes of the source tissue, including species, age, size, and anatomical location. To effectively remove cellular components, researchers have developed a variety of decellularization techniques that encompass physical, chemical, and biological/enzymatic treatments (**Figure 3A** and **Additional Table 2**), as well as combinations of these methods (Mendibil et al., 2020; Narciso et al., 2022).

**Additional Table 2:** Physical, chemical, biological, and alternative methods used for decellularization

Method	Mechanism	Application	Disadvantage	Reference
**Physical method**				
Freeze-thaw cycling	Small ice crystals form around the plasma membrane, thereby damaging the cell membrane.	Structures are relatively simple and have smaller volumes, such as skin and cornea.	Ice crystals would slightly damage the microstructure of the ECM.	Cortiella et al., 2010; Flynn, 2010
Perfusion	Inject the reagents into the intrinsic vascular system of the organ or tissue to establish a circulation pathway.	Larger and thicker tissues or entire organs.	The technique is complex, the cost is relatively high, and the removal of cells is incomplete.	Willemse et al., 2020
Immersion and agitation	Fully contact with the reagents and promote cell fragmentation.	Small and fragile structures lacking a vascular system.	Appropriate mechanical force needs to be controlled, and might cause damage to the ECM structure.	Nguyen and Tran, 2018; Alshaikh et al., 2019
**Chemical method**				
Ionic detergents (SDS, SDC)	Disruption of the phospholipid bilayer of the cell membrane leads to cell lysis.	Entire organs and denser tissues.	Destruction of the ECM structure and collagen integrity leads to a reduction in the content of GAGs.	McCrary et al., 2020
Non-ionic detergents (Triton X-100/114)	Dissolve the cell membrane and nuclear membrane and denature proteins.	Entire organs.	The cell nucleus and DNA cannot be completely removed, necessitating the use of additional methods for effective results.	Aeberhard et al., 2020; Urner et al., 2022
Zwitterionic detergents (CHAPS, SB-10/16)	Demonstrate the characteristics of ionic and non-ionic detergents.	Blood vessel and nerve tissue.	High residual DNA levels require additional washing steps to improve efficiency.	Farag et al., 2018
Hypotonic solutions Hypertonic solutions	Induce cell lysis and separating DNA from protein. Use osmotic shock to rupture the cell membrane and induce cell lysis.	Orthopedic and tendon tissues. Tendon and nerve tissues.	Failure to effectively eliminate DNA, instability of the treatment outcome, and limitations regarding complex tissue architectures.	Cheng et al., 2014; Shpichka et al., 2017
Acids Bases	Catalyze the hydrolytic degradation of biomolecules.	Thin tissues.	Disruption of the structure of the ECM and reduction of the content of GAGs and growth factors.	Poornejad et al., 2016; Hsieh et al., 2020
**Biological method**				
Trypsin	Bind to cell membrane proteins, hydrolyze into small fragments, and disrupt cell membrane integrity.	Vascular tissues.	The structural damage and the decline in mechanical stability of the ECM.	Luo et al., 2011; Li et al., 2020
Nuclease	Cleave nucleic acid sequences to promote DNA elimination while preserving proteins.	Nerve and corneal tissues.	Loss of ECM components such as hyaluronan, laminin, and collagen IV	Simsa et al., 2018; Ramm et al., 2020
**Alternative method**				
Vacuum-assisted	Accelerate decellularization by negative pressure.	Tracheal tissues and large-sized enthesis.	Increase the porosity and disrupt the collagen fiber bundles, thereby weakening the mechanical strength.	Butler et al., 2017
Apoptosis-assisted	Utilize poptosis inducers to eliminate cellular components.	Complex structures, such as neural tissues or vascular systems.	Not yet widely used.	Novoseletskaya et al., 2020; Song et al., 2021
scCO_2_	The low viscosity and high conveyance characteristics of the detergent cause cell shedding.	Corneal tissue and pulmonary arteries.	Damage to the ultrastructure of the native ECM.	Gil-Ramirez et al., 2020; Duarte et al., 2022

CHAPS: 3-[(3-Cholamidopropyl) dimethylammonio]-1-propanesulfonate; DNA: deoxyribonucleic acid; ECM: extracellular matrix; GAGs: glycosaminoglycans; SB-10: 3-(N,N- dimethyldecylammonio) propane-1-sulfonate; SB-16: 3-(N,N-dimethylhexadecylammonio) propane-1-sulfonate; SDC: sodium deoxycholate; SDS: sodium dodecyl sulfate; scCO_2_: supercritical carbon dioxide; Tris-HCl: Tris (hydroxymethyl) aminomethane hydrochloride.

#### Physical treatments

Physical methods involve the use of physical forces or processes to disrupt cell membranes and cell structures, thereby releasing and removing cellular contents. In particular, freeze-thaw cycling, soaking and agitation, as well as perfusion, are widely used in decellularization. The rapid freezing method utilizes the quick freezing of tissues to form tiny ice crystals around the plasma membrane, thereby disrupting the cell membrane (Flynn, 2010). However, a single freeze-thaw cycle involves freezing in nitrogen, followed by freeze-drying, and then thawing in a buffer solution. This process does not completely remove cell membranes and intracellular components, necessitating multiple freeze-thaw cycles (Cortiella et al., 2010). Additionally, ice crystals may slightly disrupt the microstructure of the ECM. For larger, thicker tissues or whole organs, perfusion is a common decellularization method. This technique involves injecting reagents into the organ or tissue’s inherent vascular system to establish a circulation pathway (Willemse et al., 2020). This allows the reagents to quickly enter the entire organ and evenly permeate through the natural vascular system, greatly enhancing the efficiency of decellularization.

Soaking and agitation, compared with perfusion, are more suitable for small and delicate organs or tissues that lack a vascular supply (Alshaikh et al., 2019). This method involves submerging the tissue in a decellularization agent and continuously agitating it. The effectiveness of this process is influenced by various factors, including agitation intensity, the agents used for decellularization, and the dimensions of the tissue (Nguyen and Tran, 2018). Once the tissue is soaked in the solution, agitation is performed to rupture the cells so they detach from the basement membrane, facilitating decellularization. It is important to apply appropriate mechanical force during this procedure, as excessive force may damage the ECM.

#### Chemical treatments

Chemical treatment involves the use of detergents and chemical reagents to destroy and dissolve cells, thereby achieving cell removal. This method offers several advantages, including ease of operation, rapid execution, and high efficiency. More importantly, it can selectively remove specific types of cells. However, chemical processing can have certain effects on the ECM and some signaling molecules, so attention must be paid to the processing conditions. Additionally, the applicability and tolerance of different cell lines and tissue samples should be verified (Dong et al., 2009).

Detergents encompass ionic, non-ionic, and zwitterionic types, which are soluble amphiphilic molecules capable of disrupting hydrophobic-hydrophilic interactions among molecules. By disrupting nuclear and cytoplasmic membranes, detergents can dissolve lipids and lipoproteins and dissociate DNA from DNA-associated proteins, thereby removing immunogenic cellular components (McCrary et al., 2020). Commonly used ionic detergents include sodium dodecyl sulfate (SDS), sodium deoxycholate (SDC), and dodecyltrimethylammonium bromide. It has been shown that an appropriate concentration of SDS can completely remove natural cells and genetic material, with higher concentrations resulting in lower residual DNA but decreased mechanical strength of the dECM scaffold. Conversely, lower concentrations of SDS may retain more collagen and reduce protein denaturation of the ECM, but they can also lead to increased residual DNA (Matuska and McFetridge, 2018). Unlike ionic detergents, non-ionic detergents such as Triton X-100 and Triton X-114 are milder in dissolving cell membranes and separating DNA from proteins. This is achieved by disrupting lipid-lipid, lipid-protein, and DNA-protein interactions while preserving protein-protein interactions. Due to their inability to denature proteins, non-ionic surfactants effectively clear cellular contents without compromising the structure and organization of collagen. However, their mild nature limits their efficacy in removing nuclear components and DNA (Aeberhard et al., 2020; Urner et al., 2022). Zwitterionic detergents possess both positive and negative ionic properties, allowing them to induce protein denaturation more easily. They are particularly effective in removing cell populations from thinner tissues. Therefore, to ensure the complete removal of cellular components, the use of these reagents often requires the assistance of other solutions or physical agents (Farag et al., 2018).

Hypotonic and hypertonic solutions can utilize osmotic pressure to induce cell lysis, contraction, and death, effectively eliminating cells while preserving the ECM structure (Shpichka et al., 2017). During decellularization treatment, the penetration of target tissues and/or oral tissues primarily involves alternating immersion in hypotonic and hypertonic solutions for several cycles (Cheng et al., 2014).

Acids and bases are cell removal methods that catalyze the hydrolysis and degradation of biomolecules, cytoplasmic components, and nucleic acids. Similar to detergents, they can disrupt the components and structure of the ECM. Therefore, appropriate acids and optimized concentrations should be selected. A 0.1% peracetic acid solution is considered an ideal treatment for thin tissues, as it minimally affects the structure and components of the ECM (Hsieh et al., 2020). Alkaline solutions may impact the structure of the ECM, alter mechanical and viscoelastic properties, reduce glycosaminoglycan content, and even remove growth factors from tissues upon prolonged exposure (Poornejad et al., 2016).

#### Biological treatments

Biological treatments for decellularization primarily rely on the use of enzymes and non-enzymatic agents. Non-enzymatic biological agents are compounds that can bind to metal ions and interfere with protein interactions. Common examples include ethylenediaminetetraacetic acid and ethylene glycol tetraacetic acid. These agents work by forming stable complexes with metal ions, thereby altering the structure and function of proteins. However, using chelating agents alone may not completely remove metal ions from within cells and protein interactions in the ECM. Therefore, in some applications, chelating agents are often used in combination with enzymes (de Lima Santos et al., 2020).

Trypsin is a commonly used enzyme that binds to proteins on the cell membrane and breaks them down into smaller fragments through hydrolysis. It also degrades lipids on the cell membrane, collectively disrupting the integrity of the cell membrane and leading to the separation of cellular components from the ECM. Similar to DNAases, trypsin has been widely employed because it can effectively decellularize the ECM without causing cellular toxicity (Li et al., 2020b). However, the cleavage of proteins in the ECM by trypsin may result in structural damage and alterations in the mechanical stability of the ECM (Luo et al., 2011).

Deoxyribonuclease (DNase) promotes DNA removal by cutting nucleic acid sequences while preserving proteins. DNase is typically used after detergent treatment to allow for faster penetration into tissues. However, prolonged treatment with DNase may alter the structure of the ECM, reduce mechanical stability, and lead to the loss of ECM components such as GAGs, laminin, and collagen IV (Simsa et al., 2018). To minimize damage to the ultrastructure of the ECM, appropriate enzyme concentrations, temperatures, and treatment times should be selected for decellularization processes (Ramm et al., 2020).

#### Alternative decellularization reagents and combination methods

The recently developed vacuum-assisted apoptosis induction technology not only enhances the efficiency of decellularization through negative pressure but also provides new opportunities for processing complex tissues. Moreover, it can be combined with other methods to optimize the final results (Butler et al., 2017). However, this method can disrupt the pore size and collagen pattern of the ECM, thereby affecting the mechanical integrity of the tissue. By using compounds such as camptothecin, apoptosis-inducing techniques trigger programmed cell death and offer unique advantages in the processing of neural tissues and vascular structures (Cornelison et al., 2018; Novoseletskaya et al., 2020; Song et al., 2021). Furthermore, treatments with supercritical carbon dioxide (scCO_2_) and ethanol can effectively remove DNA and reduce the phospholipid content of tissues while having minimal effects on the mechanical characteristics of tissues such as the aorta. This allows the ECM to be prepared and stored without freeze-drying. To avoid protease degradation of the ECM during the decellularization process, serine protease inhibitors such as aprotinin or leupeptin can be added to the solution (Gil-Ramírez et al., 2020; Duarte et al., 2022). The pH of the buffer solution can be maintained close to neutral, and the temperature and exposure time can be strictly controlled under certain conditions to reduce protease activity. Chen et al. (2023c) also developed a novel strategy for manufacturing acellular spinal cord fibers through electrospinning technology. Acellular spinal cord fibers can effectively retain the main protein content in spinal cord ECM and support the activity of neural progenitor cells. Although these technologies have great potential for widespread application, their high operational difficulty has prevented them from being widely used in practical applications, and they remain in the stage of in-depth research.

Physical, chemical, and enzymatic treatment methods each have their own advantages and disadvantages. While all three can remove cellular components to a certain extent, they also have the potential to damage the remaining components, structure, and performance of the ECM. Generally speaking, relying solely on any single treatment method is insufficient to minimize adverse effects on the ECM while maximizing the removal of cellular contents. Therefore, it is often necessary to combine multiple methods (Nieto-Nicolau et al., 2021). For example, the combination of freezing, drying, and ultrasonic treatment has been shown to be more effective in removing cells from fresh pig throats compared to freeze-drying alone, resulting in better outcomes (Hung et al., 2013). Additionally, researchers have developed a technique called cryochemistry, which combines physical and chemical methods for whole liver decellularization. This approach successfully yields a decellularized liver scaffold that mimics a 3D biological environment (Jiang et al., 2014).

The dECM scaffold must undergo sterilization before use to ensure its safety and reliability. Common sterilization methods include acid incubation, solvent exposure, ethylene oxide treatment, gamma irradiation, electron beam irradiation, and scCO_2_ treatment. Similar to the decellularization process, sterilization can disrupt the ultrastructure of the ECM and affect its mechanical strength and bioactive components (McDevitt et al., 2003). Therefore, it is essential to select appropriate sterilization methods based on the material and structural characteristics of the dECM scaffold. Accurate control of sterilization conditions and duration is critical to ensure the sterility of the dECM scaffold. This careful approach guarantees the safety and effectiveness of using the dECM scaffold and provides reliable support for subsequent clinical applications.

### Initial source materials for decellularized extracellular matrix grafts

The rapid advancement of tissue engineering in recent decades has captivated researchers in the field of bio-scaffolds, primarily due to their remarkable biocompatibility, bioactivity, and suitable mechanical properties (Du et al., 2018; Wu et al., 2023b; Redolfi Riva et al., 2024). Among the various types of grafts, dECM grafts have garnered significant attention. These grafts, crafted from biological materials sourced from human or animal cells, tissues, or organs, undergo decellularization processes to eliminate immunogenic cells, resulting in superior performance (Bejleri and Davis, 2019; Zhang et al., 2022c; Zhu et al., 2023). By removing immunogenic cells from homologous or heterologous tissues and organs through decellularization, dECM grafts reduce the potential for adverse host reactions and help mitigate pro-inflammatory and immune rejection responses in recipients (Kasravi et al., 2023). Additionally, dECM grafts retain the original architectural components and surface morphology of the natural ECM, along with resident ligands (Wang et al., 2023a). They not only provide effective mechanical support for nerve regeneration but also reversibly bind growth factors and cytokines, influencing macrophage activity and regulating cell migration, cell cycle progression, and cell fate (Xing et al., 2020). Furthermore, proteins in the ECM interact with cell surface receptors through integrins, forming a dynamic cell-matrix interaction network that regulates a series of downstream signaling pathways (Sainio and Järveläinen, 2020). Consequently, dECM grafts offer biomechanical and biochemical information essential for cell regeneration and tissue repair, creating a natural microenvironment that helps maintain and promote cell phenotypes adapted to their location, thereby facilitating tissue regeneration (Zhang et al., 2022c; Liang et al., 2023a; Wang et al., 2023b; Zhu et al., 2023; Sharma et al., 2024). Given the ongoing shortage of organ donors, ECM-based grafts are considered a promising alternative in neural regenerative medicine. Through decellularization methods, dECM grafts have emerged as a crucial area of research in tissue engineering and neural regenerative medicine, offering an innovative therapeutic strategy to address organ donor shortages and enhance tissue regeneration (Kim et al., 2022b; Min et al., 2022; Shi et al., 2023). Based on the origin of the ECM, dECM grafts can be categorized into two main types: tissue-derived dECM (TDM) grafts and cell-derived dECM (CDM) grafts (Zhang et al., 2022c; **Figure 3A**).

#### Tissue-derived decellularized extracellular matrix

TDM are biological substances obtained from either homologous or heterologous tissues or organs. Research has shown that the application of TDM to experimental animal models or patients in clinical trials can lead to pathogen transmission, as well as inflammatory and immune rejection reactions. To ensure biocompatibility and safety, a more intensive and systematic decellularization process is required (Choudhury et al., 2020). Typically, tissue slices or small samples serve as pre-treatment materials for the decellularization process. However, larger tissue blocks necessitate longer durations for decellularization via chemical and enzymatic reactions. Moreover, when selecting suitable decellularization agents, it is essential to comprehensively consider the cell and lipid contents, as well as the distribution characteristics of the tissue (Kabirian and Mozafari, 2020). For small, simple tissues such as the small intestine and gallbladder, decellularization can often be achieved using a single chemical bath treatment. In contrast, for solid organs such as skin, nerves, and heart valves, multiple treatments involving chemical baths and mechanical processes are frequently required to obtain decellularized tissue (McInnes et al., 2022). In the preparation of bone-based dECM, the osmotic properties of the decellularization solution must be carefully optimized to ensure the complete removal of cellular components. The use of freeze-thaw cycles is recommended in the initial stages of the decellularization process, as it not only enhances the penetration efficiency of chemical reagents but also aids in the recovery of the tissue’s ultrastructure (Arenas-Herrera et al., 2013). Additionally, demineralization and lipid removal processes are necessary for both bone and adipose tissues. Because bone tissue has a high fat content and wettability, these factors can significantly affect decellularization efficiency. Consequently, nonpolar solvents (e.g., acetone or chloroform) are often used to remove lipids initially, thereby accelerating subsequent decellularization processes. Although the preparation of TDM is complex, the resulting biodegradable biomaterials contain abundant tissue-specific bioactive molecules and complex vascular network structures, which offer significant advantages in the intricate process of tissue regeneration (Lin et al., 2020; Ullah et al., 2020). The microenvironmental signals retained in TDM provide fine structures that regulate various extracellular stromal cell interactions and guide cell growth (Safari et al., 2019). By incorporating appropriate materials or functional cells, refunctionalized TDM biomaterials can be utilized to construct tissue-engineered grafts with enhanced reparative functions.

#### Cell-derived decellularized extracellular matrix

During the culture process, cells may secrete ECM proteins to remodel the surrounding environment, making it more conducive to cell proliferation and differentiation after cell adhesion. Following decellularization treatment, these functional ECM proteins can reconstruct ECM derivatives, and this cell-free method is believed to be safer and less problematic than direct transplantation (Rani et al., 2015). Due to cell lineage functions, CDMS derived from various cell types with the same chemical composition may differ in protein concentration and type. Stem cells are considered the most beneficial cell type in the CDM production process. External changes in the cellular microenvironment can positively influence stem cells, promoting self-renewal through division and facilitating tissue renewal and regeneration (Phothichailert et al., 2024). Researchers have discovered through proteomic analysis that nerve grafts modified with BMSC-ECM induce much lower immune responses compared to nerve grafts modified with ECM from other source cells (e.g., Schwann cells, fibroblasts, Schwann cells differentiated from skin progenitor cells), proteomic compositions of which are more similar to those of autologous decellularized nerves (Wang et al., 2022a). Another report indicates that adult progenitor cells recovered from bone marrow, fat, and articular cartilage have distinct ECM protein profiles and display different chondrogenic efficiencies. The dECM scaffolds made from human adipose-derived stem cells produce a fibrous ECM pattern (high in fibronectin and type I collagen) that facilitates quicker fibrous filler formation, while human bone marrow mesenchymal stem cells and human dental pulp cells generate dECM matrices that provide a chondrogenic environment rich in aggrecan and type III collagen, promoting desirable cartilage-like tissue regeneration (Jiang and Tuan, 2023). Therefore, carefully selecting cell sources to prepare dECM based on research requirements and the characteristics of the graft can guide a method to enhance tissue regeneration.

### Various application forms of decellularized extracellular matrix grafts

The application of dECM biomaterials is extensive. They can be used directly as patches without further degradation or crushing of the structure, or they can be crushed into granular form and mixed with bioactive materials for use. Soluble dECM with good viscosity is more suitable for preparing injectable hydrogels or self-assembled bio-inks. Additionally, soluble dECM can directly encapsulate and culture cells, providing a 3D microenvironment similar to that *in vivo*, which is ideal for matching defects of any shape and size. Compared to soluble dECM, solid dECM offers several advantages in collection and processing. The mechanical strength and ECM structure of solid dECM not only maintain its physical integrity but also support re-cellularization (Li et al., 2021b; **Figure 3B**).

#### Decellularized extracellular matrix wraps

For the direct end-to-end suture of injured nerves, a nerve wrapper is essential at the anastomosis site to prevent the invasion of connective tissue and promote nerve regeneration. Decellularized membrane tissues, such as porcine small intestinal submucosa dECM and fetal porcine urinary bladder dECM, can be directly employed to wrap the nerve anastomosis site and facilitate nerve remodeling (Ren et al., 2018; Burahee et al., 2024). However, dECM wrappers derived from non-membranous tissues, such as the sciatic nerve, undergo a series of shaping processes: grinding the dECM nerve tissue into powder, dissolving the obtained powder in 0.3% acetic acid, transferring the digested solution to a culture dish, and inducing the transformation of the dECM solution into a membrane by drying at room temperature (Li et al., 2020d). Currently, commercial dECM wrappers for nerve repair surgeries are available in clinical settings. For instance, AxoGuard® nerve protector is a material similar to the nerve epineurium, created by incorporating the patient’s own cells into the dECM, and is designed to protect and isolate the nerve anastomosis site during the postoperative healing period (Suryavanshi et al., 2020; Burahee et al., 2024).

#### Decellularized extracellular matrix scaffolds

The application of dECM scaffolds derived from peripheral nerves can effectively repair peripheral nerve injuries. Researchers have successfully utilized decellularized nerve allografts to repair 2 cm sciatic nerve defects in rats using chemical decellularization techniques, achieving axonal regeneration and functional recovery (Rana et al., 2017). Similar studies have been conducted using decellularized allogeneic or xenogeneic nerve tissues, as well as non-nerve tissues, providing excellent examples of the application of tissue-derived ECM nerve scaffolds (Philips et al., 2022; Mahdian et al., 2023; Suzuki et al., 2025). Chemically decellularized allogeneic nerve allografts have successfully bridged facial nerve defects, yielding satisfactory results (Zhu et al., 2022). Researchers used scCO_2_ extraction combined with technology to treat the peripheral nerves of large mammals, such as Yorkshire pigs, to obtain decellularized xenogeneic nerve allografts for repairing sciatic nerve defects in rats. The repair results were comparable to those of autologous nerves (Wei et al., 2022). Decellularized nerve allografts have been commercialized, with Axogen’s “Avance® Nerve Graft” proving to be an effective product for nerve defect repair since its introduction (Kasper et al., 2020).

To overcome the shortcomings of tissue-derived ECM neural scaffolds, such as donor scarcity, pathogen transmission, and uncontrollable degradation kinetics, recent studies have begun to focus on cell-derived ECM scaffolds (Guan et al., 2022; Rao et al., 2022; Ding et al., 2023). These scaffolds are cultivated and obtained under pathogen-free conditions, eliminating the risk of pathogen transmission while maintaining the required geometry and flexibility when recombined with synthetic or naturally derived biomaterials. Our research group, led by Xiaosong Gu, used BMSCs, Schwann cells, Schwann cells derived from skin precursor stem cells, and fibroblast-derived dECM-modified chitosan/silk conduits to repair sciatic nerve defects in rats, achieving excellent repair outcomes (Xue et al., 2017; Zhu et al., 2018; Wang et al., 2022a). Through proteomic analysis, we found that nerve grafts modified by BMSC-ECM significantly reduced the induced immune response compared with nerve grafts modified by ECM from other cell sources (such as Schwann cells, fibroblasts, and Schwann cells differentiated from skin-derived progenitor cells). Additionally, BMSC-ECM exhibited similar components and spatial structural characteristics to decellularized nerves, forming a microenvironment conducive to the growth of nerve cells and promoting nerve regeneration both *in vivo* and *in vitro* (Wang et al., 2022a). Our team further used a structurally complete and bioactive multi-layer hBMSC-dECM to wrap the PLGA fiber scaffold, combined with degradable nerve conduits, successfully constructing a 3D oriented nerve graft based on the cell matrix. The results indicate that the unique biochemical and structural components present in the cell matrix-modified nerve grafts play a crucial role in promoting initial cell adhesion and exerting temporal and spatial control over the microenvironment to stimulate and enhance nerve regeneration (Wang et al., 2024b).

To better maximize the capabilities of electrospun nerve scaffolds, researchers have investigated the use of synthetic polymers combined with dECM. Hybrid electrospun scaffolds incorporating dECM have been developed using electrospinning blending technology (Politi et al., 2020; Brown et al., 2022). Schwann cells were applied to D,L-PLGA-silk fibroin-collagen scaffolds, which were fabricated through electrospinning blending methods. Experimental results indicate that, compared to pure PLGA scaffolds, the PLGA-SF-COL scaffolds exhibit superior properties for nerve tissue engineering (Wang et al., 2011a). Additionally, surface coupling techniques have been employed to coat the surfaces of electrospun phases with dECM particles (Masaeli et al., 2017). Due to their exceptional mechanical performance, biomimetic microarchitecture, and diverse inventory of bioactive molecules, electrospun scaffolds have been experimentally shown to effectively support peripheral nerve regeneration.

#### Decellularized extracellular matrix microparticles

While dECM scaffolds enable the fabrication of 3D constructs that impart biomechanical cues, tunable dECM microparticles created by freeze-drying and pulverizing tissues exhibit unmatched versatility for use in tissue engineering. These microparticles can be tailored in geometry to fit and fill irregularly-shaped defects, allowing for the consideration of minimally invasive implantation approaches (Capella-Monsonís et al., 2024). In addition, the production of microparticles allows for the customization of size, solubility, and cross-linking properties with high accuracy to achieve optimal functionalities. Furthermore, dECM microparticles have been employed to prepare dECM “paper” by transforming the suspended microparticles into ink and shaping the ink using specific casting and drying techniques. It has been demonstrated that this dECM “paper” can effectively support the adhesion, survival, and proliferation of heterogeneous mesenchymal stem cells derived from human tissues. Furthermore, suspending the microparticles in synthetic or natural gels may better maintain the biophysical properties of the ECM compared to fully solubilized soluble dECM (Spang and Christman, 2018). However, attention should be given to the particle size and concentration of the microparticles in actual applications. For example, ultrafine dECM microparticles with sizes ranging from 1 to 50 μm biodegrade more quickly than micro dECM microparticles with sizes ranging from 100 to 1000 micrometers (Wang et al., 2021).

#### Decellularized extracellular matrix hydrogels

The dECM is freeze-dried, ground into powder, digested with pepsin, and then formed into a 3D network through physical cross-linking or self-assembly of collagen fibers (Kort-Mascort et al., 2023). After dissolution, it is adjusted to physiological pH and temperature to prepare a homogeneous and soluble dECM hydrogel that undergoes thermogelation at physiological temperature and pH (Yeleswarapu et al., 2022). ECM-derived hydrogels are used to deliver soluble factors, such as growth factors and biological agents, and to increase the retention rate of transplanted cells (Vriend et al., 2022). Hydrogels based on porcine nerve-derived dECM have been fabricated and shown to promote axon growth effectively. Schwann cells were observed to grow around the neurites in *in vitro* experiments (Kuna et al., 2022). Due to their physical properties and ability to promote Schwann cell proliferation, dECM-based hydrogels are often used as lumen fillers for nerve conduits or as carriers for Schwann cell transplantation (Gregory et al., 2022; Wang et al., 2022b; Kellaway et al., 2023). In a 15 mm sciatic nerve defect rat model, a combination therapy using a pDNM-based hydrogel along with electrospun polylactic acid-polycaprolactone conduits resulted in improved repair without eliciting an immune response associated with M2 macrophages related to constructive remodeling reactions. Morphological analysis and electrophysiological and functional tests indicated the same regenerative effect as that observed when using rat acellular nerve matrix allograft scaffolds (Lin et al., 2018b). The nanofiber scaffold, consisting of neatly aligned polylactic acid nanofibers decorated with 0.25% pDNM gel, offers topological and biochemical guidance for directing and facilitating axonal extension, myelination of nerve fibers, and functional restoration (Zheng et al., 2021). Hydrogels have emerged as a new type of biodegradable and biocompatible matrix material capable of carrying and delivering multiple factors crucial for axonal regeneration and neuronal survival, such as glial cell line-derived neurotrophic factor (Fan et al., 2025). Although dECM-based hydrogels are highly malleable, they are more prone to losing the natural structure of the ECM due to changes in the ultrastructure of proteins. Moreover, because of their high water content and open usage environment, they are more susceptible to bacterial contamination.

#### Three-dimensional bioprinting inks derived from decellularized extracellular matrix

While traditional pure injectable dECM hydrogels cannot achieve spatial stability, spatially stable hydrogels can be established using 3D bioprinting technology, which provides more accurate control over the deposition of dECM biomaterials as well as cell spatial localization (Das et al., 2019; Kim et al., 2020). It is important to note that the prepared dECM-based bioink needs to not only have good compatibility with the cells but also meet the processability requirements for the fabrication of 3D cell-laden constructs. During the decellularization process, the destruction of ECM proteins leads to dECM bioinks having lower viscosity, which limits their performance in bioprinting (Pati et al., 2014). To address this, researchers have incorporated biomaterial additives, including alginate, silk proteins, and gelatin, into dECM bioinks, which have significantly enhanced the printing conditions and cell viability (Lee et al., 2020). Additionally, the bioprinting process relies on the cross-linking properties of materials to achieve the transition from the sol state to the gel state. A dual-network cross-linked bioink formulated from a mixture of methacrylated hyaluronic acid and dECM derived from porcine articular cartilage can be cross-linked under visible light and at 37°C, respectively. dECM added at a concentration of 10 mg/mL has been shown to effectively enhance the mechanical properties and cell survival rate of the material (Li et al., 2022a).

Moreover, 3D printing technology has recently been applied to the field of peripheral nerve repair (Liu et al., 2022c). An innovative 3D-printed polycaprolactone conduit was developed, with the surface of the tube coated with a composite of dECM and polydopamine. This composite-covered tube exhibits not only superior mechanical and hydrophobic properties but also significantly enhances the adhesion, proliferation, and differentiation of Schwann cells. Additionally, it markedly increases the expression of cell-specific neural markers such as Nestin, TUJ-1, and microtubule-associated protein 2 (Chen et al., 2018). Furthermore, research groups have successfully engineered 3D electrospun nanofiber scaffolds coated with native extracellular matrix. These scaffolds demonstrate well-defined controllable alignment patterns that sufficiently replicate the characteristics of human tissues. Importantly, they not only exhibit a series of improved functional aspects for cells but also significantly reduce the potential for immune responses (Sharma et al., 2024).

### Cellular events associated with the regeneration process mediated by decellularized extracellular matrix grafts

ECM contains multiple ECM-matrix proteins, including core matrix proteins (such as glycoproteins, collagens, and proteoglycans) and matrix-associated proteins (such as regulators, secreted factors, and ECM-related proteins). Single or multiple ECM molecules serve as key chemical signals in the tissue microenvironment, regulating cell differentiation, lineage fate choice, and other cell-tissue interactions (Zhang et al., 2024a). The assembled dECM can activate and orchestrate multiple intracellular signaling cascades, prompting cells to generate various reactions and regulate their behavior (Smoak et al., 2021). A comprehensive investigation of the complex cellular responses associated with dECM biomaterials *in vivo*, including inflammation, cell adhesion, proliferation, and differentiation, is of great significance, as it will provide fundamental support for the fabrication of dECM biomaterials that mimic specific defect microenvironments.

#### Decellularized extracellular matrix grafts enhance cell adhesion

Regarding cell adhesion, proteins and peptides within the dECM graft bind to integrin receptors to promote cell attachment to the graft. Integrin receptors act as biosensors on the cell surface and are responsible for transmitting signals between the inside and outside of the cell. This adhesion provides the mechanical support necessary for cell stability and function (Phothichailert et al., 2024). Focal adhesions are large molecular complexes composed of ECM, integrins, the intracellular cytoskeleton, and other proteins, including kindlin, talin, vinculin, paxillin, PINCH, Src, focal adhesion kinase, and integrin-linked kinase. As mechanical links between the ECM and the cytoskeleton, focal adhesions play a crucial role in mediating communication between cells and their environment, regulating important processes such as cell adhesion, spreading, migration, differentiation, and mechanotransduction by influencing various outside-in and inside-out signaling pathways (Chen et al., 2023a; Huber et al., 2023). Collagen, laminin, and fibronectin in the dECM graft can interact with 11 types of integrins. These interactions ensure cell adhesion to the ECM and facilitate information transmission (Myers et al., 2011). dECM derived from human bone marrow mesenchymal stem cells may promote initial cell adhesion and guidance during the process of nerve regeneration by activating the PI3K-Akt signaling pathway (Wang et al., 2024b).

#### Decellularized extracellular matrix grafts enhance cell proliferation and differentiation

A series of bioactive molecules in the dECM scaffold, such as ECM proteins, growth factors, and cell adhesion proteins, can modulate cell proliferation and differentiation by providing biochemical signaling and mechanical support. Fibroblasts exhibit relatively high proliferation levels on the fibronectin matrix, while they show lower proliferation levels on the laminin matrix. However, the proliferative response of epithelial cells to fibronectin and laminin matrices is the opposite (Dalton and Lemmon, 2021; Karamanos et al., 2021). Additionally, ECM components, including laminin, fibronectin, and collagen, can significantly support the proliferation of Schwann cells *in vitro* and lead to an increase in the number of neurites per cell, indicating that these ECM components promote the secretion of neurite growth factors in Schwann cells (Jiang et al., 2024b; Pinzon-Herrera et al., 2024). For stem cells, the physiological balance between their quiescent and proliferating states can be disrupted by exposure to oxidative stress caused by inflammation, which may lead to premature aging and weaken their regenerative potential (Rochette et al., 2020). It has been confirmed that type I collagen in decellularized matrices can partially alleviate the premature senescence of stem cells by triggering SIRT1-dependent signaling pathways. Even when stem cells still exhibit senescence phenotypes, their differentiation ability in osteogenic differentiation can be significantly enhanced (Zhou et al., 2018). Furthermore, fetal heart-derived dECM can induce proliferative reprogramming of cardiomyocytes (Bejleri and Davis, 2019) and stimulate the cell cycle of cardiomyocytes by increasing nuclear localization of YAP (Wang et al., 2020).

Multiple studies have evaluated the effect of dECM grafts on stem cell differentiation and lineage fate. Certain matrix-binding proteins can serve as markers for stem cell surface proteins. For instance, CD49a-f is expressed on the surface of basal epithelial progenitor cells and tumor stem cells. CD29 is highly expressed in neural stem cells, but its expression decreases as they differentiate into neurons (Niklason, 2018). Both *in vitro* and *in vivo* studies have shown that periodontal ligament stem cells exhibit enhanced proliferation and osteogenic differentiation in dECM rich in COL4A2, and this effect is achieved through negative regulation of the canonical Wnt/β-catenin signaling pathway (Wen et al., 2019). Meanwhile, the dECM grafts also provide a microenvironment for stem cell differentiation. For example, laminin constitutes the ECM niche of trophoblast stem cells *in vivo*, and collagen and laminin are the ligands of the trophoblast stem cell niche (Kiyozumi et al., 2020).

#### Regulatory effect of decellularized extracellular matrix grafts on immune-related cells involved in inflammation suppression

During implantation, the immune response orchestrated by various immune cells (e.g., macrophages, T cells, and mast cells) significantly affects the regeneration outcome of dECM grafts (Chakraborty et al., 2020). Due to their environment-conditional phenotypic features, macrophages play a vital role in the immune system owing to their specific functional plasticity. Generally, macrophages that initially accumulate at the inflammation site may polarize to the M1 phenotype to facilitate antigen phagocytosis, degradation, and activation of tissue-resident fibroblasts. In contrast, M2 macrophages, which play a regenerative role, recruit stem cells, induce angiogenesis, and secrete cytokines that counteract inflammation (Kadomoto et al., 2021; Chen et al., 2023b). Consequently, most research on dECM-based studies aims to achieve effective regulation of the immune system by promoting the switch from M1 to M2 phenotype. It has been shown that dECM-based biomaterials can encourage the polarization of M2 macrophages during the early stages of injury and reduce the M1/M2 ratio, thereby promoting neuronal growth, chondrogenic differentiation, and osteoinductivity (Hong et al., 2020; Zeng et al., 2022; García-García et al., 2023). However, premature modulation from M1 to M2 may not always benefit tissue regeneration (Long et al., 2023). Therefore, future studies should explore how dECM can dynamically influence this shift.

Concerning T cells, the activation of sensitized T cell proliferation and transformation to the Th2 phenotype by dECM has been reported, primarily associated with the regenerative mechanism (Ozpinar et al., 2021). The ultrastructure of dECM proteins and their level of cross-linking play an important role in the regulatory function of helper T cells in the immune response (Chakraborty et al., 2020). Regarding mast cells, there have been few related studies due to the lack of suitable *in vivo* models. One earlier study used dECM hydrogel extracted from porcine dermis as a 3D culture platform to investigate the biological characteristics of human mast cells. The results demonstrated that, compared to type I collagen, human mast cells cultured in dECM hydrogels exhibited high metabolic activity, strong cell viability, and increased expression levels of IgE receptors, which are closely related to mast cell maturation (Ozpinar et al., 2021). Additionally, fibroblasts can also interfere with immune responses via dECM interaction. After dECM derived from WI-38 human lung fibroblasts was introduced into a collagen-based bioactive scaffold, the mRNA level of the pro-inflammatory matrix metalloproteinase TIMP2 in skin fibroblasts decreased, while the expression level of matrix metalloproteinase MMP3 increased sharply (Kim et al., 2022a).

### Advantages and disadvantages of decellularized extracellular matrix grafts

**Figure 4:**
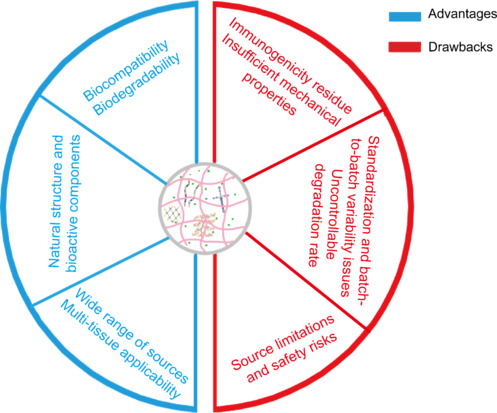
The advantages and drawbacks of decellularized extracellular matrix grafts. The advantages are highlighted in blue, while the drawbacks are indicated in red.

Over the past decade, dECM biomaterials have garnered considerable attention and sparked intense research in the fields of tissue engineering and regenerative medicine. Originating from cells, tissues, and organs, these biomaterials have had their therapeutic potential affirmed (Amirazad et al., 2022; Kim et al., 2022a; McInnes et al., 2022; Quinteira et al., 2024). FDA-approved grafts, such as demineralized bone, skin, and ligaments, have further demonstrated their clinical value as tissue substitutes (Patel et al., 2021; Cifuentes et al., 2024). Ongoing research has optimized the methods and protocols for developing non-immune biomaterials derived from cells and tissues, endowing them with reproducible structures and biological functions. dECM, with its versatile nature, enables the development of various biomaterial forms, including powders, gels, thin sheets, 3D structures fabricated through additive manufacturing, and even tissue and organ structures featuring intact vascular architecture and neural innervation (Mahara et al., 2022; Li et al., 2024a; Wang et al., 2024a). Due to their innate structural and compositional attributes, which encompass binding motifs and biochemical signaling cues, these non-immune and bioactive biomaterials outperform engineered matrices made from natural or synthetic materials in terms of performance. They play a pivotal role in tissue repair and regeneration by facilitating host cell interactions and guiding tissue regeneration (Li et al., 2022c; Liu et al., 2022a; Stone et al., 2023; Qiao et al., 2024). Additionally, dECM also serves as a drug delivery carrier to achieve controlled release of bioactive molecules (Kolliopoulos and Mikos, 2025). The specific advantages are as follows (Massaro et al., 2021; Zhu et al., 2021; Sarmin et al., 2022; Cui et al., 2025; **Figure 4**).

Outstanding biocompatibility: By eliminating cellular components, such as DNA and lipids, this approach significantly reduces immunogenicity and minimizes host rejection reactions. Consequently, it is well-suited for allogeneic or xenogeneic transplantation.

Preserve natural structure and bioactive components: Maintain the natural 3D structure and porosity of the ECM to promote cell migration, adhesion, and angiogenesis. Retain components such as collagen, fibronectin, and growth factors to provide microenvironmental signals that support cell proliferation and differentiation.

Multi-tissue applicability: It can be used for repairing various tissues, including skin, heart, liver, and skeletal muscle, and it supports the directed differentiation of stem cells.

Biodegradability: The stent is gradually absorbed by the body, thereby eliminating the necessity for a second surgical procedure. Furthermore, the degradation products are non-toxic.

Wide range of material sources: They can be sourced from human tissues (such as placenta and dermis) or animal tissues (including porcine small intestinal mucosa and bovine pericardium), thereby addressing a variety of needs. Despite the remarkable achievements, there are still some drawbacks (Liao et al., 2020; Barbulescu et al., 2022; Brown et al., 2022; **Figure 4**).

Potential immunogenic residues: Incomplete decellularization processes may lead to the retention of cell debris or antigens (such as α-gal antigen), causing chronic inflammation or rejection reactions.

Insufficient mechanical properties: The mechanical strength of natural ECM is relatively limited, especially in load-bearing areas such as bones and joints, where it is prone to structural failure. Therefore, it is often necessary to combine natural ECM with synthetic materials.

Standardization and batch-to-batch variability issues: The inherent variability in composition and structure due to natural origins presents significant challenges in achieving consistency during large-scale production, which in turn affects clinical reproducibility.

Uncontrollable degradation rate: The disparity between the degradation rate and the tissue regeneration rate can lead to repair failure if degradation occurs too rapidly or provoke foreign body reactions if it proceeds too slowly.

Source limitations and safety risks: Human-derived materials are limited in availability, while animal-derived materials require rigorous pathogen treatment, such as addressing porcine endogenous retroviruses. This necessity increases both the cost and complexity of their preparation (Klak et al., 2021; Li et al., 2021b).

## Safety Considerations of Decellularized Extracellular Matrix Scaffolds: Immunogenicity, Toxicity, Degradation Kinetics, and Peripheral Nerve Repair

### Immunogenicity and host response

The primary objective of decellularization is to eliminate cellular components to minimize immunogenicity. Incomplete decellularization can trigger TLR9-mediated immune responses, leading to macrophage infiltration, chronic inflammation, fibrosis, or graft rejection (Suresh et al., 2016). In pDNMs, the presence of α-Gal epitopes poses a significant immunogenic risk. Residual α-gal antigens can enhance B-cell and T-cell responses, increasing the likelihood of rejection, whereas treatment with α-galactosidase effectively mitigates this risk. In contrast, human-derived decellularized nerve matrices exhibit lower immunogenicity due to reduced MHC-I expression, resulting in milder host responses. For xenogeneic scaffolds, such as pDNMs, combinatorial decellularization strategies are necessary to achieve biocompatibility comparable to that of human-derived decellularized nerve matrices (Li et al., 2023b).

### Potential toxicity of decellularization agents

Chemical detergents and enzymes are commonly used to eliminate cellular debris, but they can damage the native ECM ultrastructure and leave behind cytotoxic residues (Ramm et al., 2020). For example, residual SDS can inhibit Schwann cell proliferation, induce membrane disruption through surfactant activity, and lead to cytoplasmic leakage and apoptosis. Highly concentrated anionic detergents can further harm cells by denaturing membrane proteins and lipids (Lee et al., 2019). Similarly, harsh physical treatments can alter tissue mechanics. Decellularized porcine kidneys subjected to uncontrolled freeze-thaw protocols have been shown to lose elastic modulus and structural integrity, potentially disrupting guidance for axonal regeneration (Poornejad et al., 2015).

### Degradation kinetics and long-term effects

Decellularized scaffold degradation kinetics should be directly correlated with the nerve regeneration process. Premature scaffold disassembly can occur with rapid degradation, while delayed degradation can lead to retraction, hindering reinnervation and eliciting a chronic foreign-body response (Liang et al., 2023b). Collagen degradation products can induce fibrosis, and improper breakdown of GAGs can disrupt neurotrophin regulation, thereby limiting functional recovery (Huynh et al., 2019).

### Safety assessment in peripheral nerve repair

Biocompatibility is a key issue in the safety evaluation of dECM grafts. On one hand, their natural material supports neuronal regeneration; on the other hand, their animal origin may lead to short-term local inflammatory reactions. These reactions can be mitigated through processing methods designed to suppress inflammation (Luo et al., 2023; Jiang et al., 2024a). Main concerns regarding toxicity focus on persistent processing reagents and degradation metabolites, which can be addressed through effective decellularization and washing procedures (Tabatabaei Rezaei et al., 2025). Regarding long-term safety, animal studies to date have shown that toxicity and carcinogenic activity are very low. However, clinical evidence concerning long-term effects remains scarce, raising concerns about integrity and degradation kinetics (Brown et al., 2022; McInnes et al., 2022). Existing assessment strategies include *in vitro* toxicology and immune response evaluations, *in vivo* toxicity (acute and chronic) and behavioral tests, post-clinical adverse event monitoring, and multi-year follow-ups in humans. Nonetheless, challenges remain in achieving manufacturing homogeneity, managing immunogenicity, and linking degradation rates to the time scale of tissue regeneration, which are significant bottlenecks for clinical translation (Barbulescu et al., 2022; Zhang et al., 2023b). These areas warrant further research to advance the development of this field.

### Current Status of Clinical Translation

We conclude that dECM grafts have made significant progress in clinical translation for peripheral nerve regeneration, advancing from the basic research stage to industrialization and clinical utilization. Notable breakthroughs include Axogen’s Avance® nerve graft, which achieved similar functional recovery to autografts in traumatic defects during phase III clinical trials and is currently under Fast Track BLA review with the Regenerative Medicine Advanced Therapy (RMAT) designation. Additionally, the decellularized matrix peripheral nerve repair membrane in China (NMPA 20193130355) has been clinically used in over thirty hospitals nationwide, demonstrating substantial clinical and socioeconomic benefits. At the Department of Laboratory Medicine of the First Affiliated Hospital, Sun Yat-sen University, it was shown that dECM grafts significantly inhibited the sprouting of neuromas following sciatic nerve transection and successfully guided the sprouts of nerve stumps into fibro-tissue structures that do not produce new sprouts (Qiu et al., 2023). Worldwide research is underway to fund the development of process improvements and the extension of indications for dECM grafts. Our key strategies are (1) regulating M2 macrophage polarization through combinatorial therapy with acellular scaffold-conditioned Treg medium to assist in creating a regenerative microenvironment in diabetic neuropathy; and (2) engineering novel 3D-printed dECM/polycaprolactone composite scaffolds to overcome biomechanical compatibility issues in critical-size defect repair.

## Current Challenges and Limitations

Although this review summarizes the latest developments and clinical translation of dECM scaffolds in peripheral nerve regeneration, several limitations must be considered. We prioritized literature research on high-impact English papers published in the past ten years; consequently, some non-English studies and emerging regional clinical applications may not be included. Decellularization methods encompass traditional approaches, as core concepts such as solvent selection, “elution” factors, and structural and mechanical supports remain common across all regeneration processes. However, emerging methods, such as scCO_2_ decellularization, require further in-depth exploration regarding their potential mechanisms and safety. Additionally, most available information in current clinical data comes from small single-center studies, while multi-center cohorts and real-world data are still insufficiently detailed. This limitation may restrict the generalizability of the results. Lastly, from the perspective of scalable production costs and the regulatory challenges that future interdisciplinary work involving technology transformation will need to address, the review of key engineering economic issues related to scaffold industrialization still requires further improvement.

## Conclusions and Future Perspectives

The recent advancements in tissue-engineered nerve grafts have introduced new approaches to managing peripheral nerve injuries. This nerve grafting method utilizes nerve scaffolds instead of Schwann cell basal lamina, providing the necessary physical and mechanical support as a bridge to facilitate axonal sprouting from the proximal nerve stump to the distal nerve stump. Additionally, nerve scaffolds secrete neurotrophic factors and transmit biochemical signals; they also exhibit adequate biocompatibility and controllable biodegradability (Swider et al., 2018; Fujimaki et al., 2019; Gu, 2022).

Although tissue-engineered nerve grafts have made significant progress in recent years, the clinical effectiveness of nerve scaffolds still requires improvement. Continuously updated and improved decellularization technology enhances the properties of dECMs, offering multiple benefits for nerve scaffolds. First, dECM scaffolds derived from decellularization eliminate immunogenic cells, thereby reducing the risk of poor immune responses from the host after grafting. Second, biomimetic scaffolds maintain the stable physical structure and signaling environment of native organs or tissues, playing a key role in modulating cell–cell and cell–ECM interactions (Lin et al., 2018a). Third, compared to synthetic polymers, dECM scaffolds provide intrinsic biological and biochemical components on their surfaces, including cell adhesive ligands (Chaudhari et al., 2016). dECMs have gained increasing prominence as strong candidates for immunocompatible organ and tissue replacement, opening new avenues for tissue repair and promoting regeneration and remodeling (Rowley et al., 2019; Taemeh et al., 2020).

There are three primary challenges to fully leverage the clinical promise of these technologies: (1) technology enhancement, including low-toxicity decellularization processes and functional upgrades; (2) clinical fine-tuning, designed for injury conditions with mechanical gradients and incorporating multimodal evaluations, including electrical, imaging, and molecular biomarkers; and (3) ecosystem building for translation, which we believe necessitates interdisciplinary cooperation to shift the paradigm toward “functional restoration” and establish a global clinical data repository to accelerate personalized applications.

Over the next decade, iterative technological refinement and rigorous clinical validation could position dECM grafts as a standard therapy for peripheral nerve repair, with the potential for expansion to complex indications such as diabetic neuropathy. Ultimately, these advances may bridge the gap from structural reconstruction to functional recovery, marking a pivotal leap in regenerative medicine.

## Additional files:

***Additional Table 1:*** *Major protein components of decellularized extracellular matrix.*

***Additional Table 2:*** *Physical, chemical, biological, and alternative methods used for decellularization.*

## Data Availability

*All relevant data are within the paper and its Additional files*.
